# Experiences and Lessons From Polio Eradication Applied to Immunization in 10 Focus Countries of the Polio Endgame Strategic Plan

**DOI:** 10.1093/infdis/jix047

**Published:** 2017-07-01

**Authors:** Maya M. V. X. van den Ent, Apoorva Mallya, Hardeep Sandhu, Blanche-Philomene Anya, Nasir Yusuf, Marcelline Ntakibirora, Andreas Hasman, Kamal Fahmy, John Agbor, Melissa Corkum, Kyandindi Sumaili, Anisur Rahman Siddique, Jane Bammeke, Fiona Braka, Rija Andriamihantanirina, Antoine-Marie C. Ziao, Clement Djumo, Moise Desire Yapi, Stephen Sosler, Rudolf Eggers

**Affiliations:** 1 Program Division, UNICEF Headquarters, New York, New York;; 2 Polio Team, Bill and Melinda Gates Foundation, Seattle, Washington;; 3 Global Immunization Division, CDC, Atlanta, Georgia;; 4 WHO Africa Regional Office, Brazzaville, Congo;; 5 UNICEF East and Southern Africa Regional Office, Nairobi, Kenya;; 6 UNICEF West and Central Africa Regional Office, Dakar, Senegal;; 7 UNICEF Regional Office for South Asia, Kathmandu, Nepal;; 8 WHO Eastern Mediterranean Regional Office, Cairo, Egypt;; 9 UNICEF Nigeria Country Office, Abuja, Nigeria;; 10 WHO Nigeria Country Office, Abuja, Nigeria;; 11 UNICEF DRC Country Office, Kinshasa, Democratic Republic of Congo;; 12 UNICEF Chad Country Office, N'Djamena, Chad;; 13 WHO DRC Country Office, Kinshasa, Democratic Republic of Congo;; 14 Gavi, the Vaccine Alliance, Geneva, Switzerland;; 15 WHO Kenya Country Office, Nairobi, Kenya; and; 16 UNICEF Afghanistan, Afghanistan

**Keywords:** Immunization, polio eradication, system strengthening, routine immunization, equity, coverage.

## Abstract

Nine polio areas of expertise were applied to broader immunization and mother, newborn and child health goals in ten focus countries of the Polio Eradication Endgame Strategic Plan: policy & strategy development, planning, management and oversight (accountability framework), implementation & service delivery, monitoring, communications & community engagement, disease surveillance & data analysis, technical quality & capacity building, and partnerships. Although coverage improvements depend on multiple factors and increased coverage cannot be attributed to the use of polio assets alone, 6 out of the 10 focus countries improved coverage in three doses of diphtheria tetanus pertussis containing vaccine between 2013 and 2015. Government leadership, evidence-based programming, country-driven comprehensive operational annual plans, community partnership and strong accountability systems are critical for all programs and polio eradication has illustrated these can be leveraged to increase immunization coverage and equity and enhance global health security in the focus countries.

Routine immunization services reached 86% of the world’s infants with 3 doses of diphtheria tetanus pertussis (DTP)-containing vaccines in 2015. Nevertheless, 19.4 million children remained unprotected in 2015 [1]. In parts of the world where polio virus circulation has been persistent or polio outbreaks have occurred, routine immunization remains particularly low. These areas tend to be part of countries or regions with weak health systems and account for a substantial portion of the world’s underimmunized children [1, 2]. More than half of the children who did not receive DTP3 live in 3 countries: India (3.2 million), Nigeria (2.9 million), and Pakistan (1.4 million). Between 2013 and 2015, India reduced the number of children underimmunized with DTP3-containing vaccine by 1 million, and Nigeria reduced the number by 0.6 million, whereas Pakistan did not make substantial progress in reducing the number of underimmunized children [1].

The Global Vaccine Action Plan aims at a world free of polio, outlines regional disease-specific elimination targets, and seeks routine immunization coverage of 90% at the national level and at least 80% coverage in every district [3]. The Reaching Every District (RED) approach has been introduced in the Expanded Program on Immunization (EPI) to increase routine immunization coverage. It outlines 5 components: (1) establishment of regular outreach or reaching of target populations, (2) supportive supervision, (3) community links, (4) monitoring and use of data for action, and (5) planning and management of resources [4]. This approach has demonstrated results [5, 6, 7], but countries struggle to implement all of the components systematically.

There have been cases where intense focus on polio eradication has undermined routine immunization programs, notably in endemic countries with active poliovirus circulation [8, 9]. Polio resources and assets, however, typically support broader immunization systems when not engaged in priority polio activities [10, 11]. For example, in the Pan American Health Region, polio eradication efforts have played a key role in driving immunization system strengthening. India’s polio program gradually increased support to routine immunization after the country was declared free of wild polio virus in 2011 and the infrastructure had more time to dedicate to wider immunization goals. Encouraged by the Indian experience in using polio assets for routine immunization strengthening, the Polio Eradication Endgame Strategic Plan 2013–2018 (PEESP) calls for polio expertise to be applied to strengthening different components of routine immunization, in particular in 10 focus countries where systems remain weak and important numbers of polio assets still exist [7].

The second objective of the PEESP calls for “strengthening immunization services” in “focus countries” and “introducing injectable inactivated polio vaccine (IPV)” and “withdrawal of oral polio vaccine (OPV) globally.” The plan outlines 3 indicators for the strengthening of immunization services in the 10 focus countries: the existence of a single annual plan encompassing broader immunization goals, the percentage of time spent by polio-funded field personnel on immunization systems strengthening, and an increase in DTP3 coverage in polio high-risk districts [7].

Although it was recognized that there was a potential contribution of polio assets to routine immunization, the lack of systematic use of polio expertise for routine immunization strengthening created a need to systematize and document these efforts. Professionals in routine immunization and polio eradication identified 9 expertise areas of polio eradication that the focus countries could explore systematically: policy and strategy development, planning, management and oversight (accountability framework), implementation and service delivery, monitoring, communications and community engagement, disease surveillance and data analysis, technical quality and capacity building, and partnerships [12, 13]. Initially, these expertise areas were applied in limited-scale projects. Subsequently, the Routine Immunization subgroup of the Immunization Management Group adopted a broader approach encouraging countries to develop a single EPI plan integrating the support provided by polio assets with specific actions focused in high-risk districts to raise coverage.

This article analyzes the application of polio expertise areas across the 10 countries and provides country examples of successes and challenges reported in the use of polio assets in routine immunization.

## POLIO EXPERTISE AREAS APPLIED TO IMMUNIZATION

Focus countries used polio assets in a number of ways to improve routine immunization coverage, surveillance, and wider health programs, although these expertise areas had been used by national immunization programs well before the rapid increase in the Global Polio Eradication Initiative’s (GPEI) direct investment several years ago. A summary of the use of polio eradication expertise areas in immunization in the 10 focus countries is provided in Table 1.

**Table 1.** Use of Polio Eradication Expertise Areas in Immunization Programs in the 10 Focus Countries

Country Coverage	Policy and strategy	Planning	Management & oversight	Implementation & service delivery	Monitoring & Evaluation	Communication and community engagement	Surveillance & data analysis	Capacity building	Partnership
Angola DTP3 2013: 77% DTP3 2015: 64%	RED activities in polio high-risk districts	Annual EPI planning and review process Microplanning	ICC for immunization and mother, newborn, child health promotion	Timely and sustainable vaccine supply Filling cold chain gap at district level Technical and supervisory support during preparation and implementation of intensification of routine immunization		Defaulter tracing	Data quality assessments Integrated disease and response surveillance (polio, measles, yellow fever, neonatal tetanus)	MLM training of district management teams RED training of frontline health workers Training on vaccine management	
Chad DTP3 2013: 48% DTP3 2015: 55%	RED activities in polio high-risk districts Special vaccination for island and nomads population (integrated with other interventions)	Annual EPI planning and review process Micro-planning	Monthly meetings with head of state expanded to EPI and health Hub coordination structure used for EPI, including monthly review meetings Social mobilization coordination meetings Compilation and feedback of process indicators for action	Vaccine management and monitoring Organization of immunization fixed and outreach sessions Organization of PIRI targeting special populations	Monitoring of process and outcome indicators at health facility level: stock out, micro-plans, sessions conducted, supervision, data quality, coverage, drop-out	Work with and train community committees Active defaulter tracing Registration of all children aged <2 y with immunization status for mobilization of unvaccinated children	Weekly visits to high-priority sites Integrated disease and response surveillance (polio, measles, yellow fever, neonatal tetanus)	MLM training for EPI and district managers, Immunization in practice (IIP) for health center staff Use of DVD-MT at district and regional levels	
DRC DTP3 2013: 74% DTP3 2015: 81%	RED activities in polio high- risk districts Negotiation with armed forces for access (North Katanga)	Annual EPI planning and review process Microplanning in all health facilities, including all villages	Weekly meeting of Strengthening the Routine Immunization group to monitor the implementation of the plan Monthly meeting of review cut off of analysis and validation of immunization data at all levels Monthly teleconference with high- risk provincesNational Coordination Committee for Health and Epidemics		Monitoring of process and outcome indicators at health facility level: vaccine stocks, kerosene availability, sessions, supervision, coordination meetings held (HF, DS), Number of undervaccinated children Monitoring tools for polio national immunization days (NIDs) (lot quality assessments) adapted for RI	Evidence generation through high quality KAPB (Harvard Polling) Involvement of community leaders in planning Mobilisation of children and women due for vaccination Sociological evidence generation on vaccine hesitancy Promotion of family key practices	Weekly visits to high-priority sites Integrated disease and response surveillance (polio, measles, yellow fever, neonatal tetanus) Ebola surveillance and response Laboratory for polio expanded to measles, rubella, yellow fever Data management training workshop and mapping	Planning Cold chain and logistics DVD-MT Placement of consultants and STOP teams in priorities antennas	Trans-border coordination Resource mobilization through Congolese parliamentarians for wider immunization
Ethiopia DTP3 2013: 72% DTP3 2015: 86%	RED activities in polio high-risk districts Use of African Vaccination Week and supplementary immunization activities for providing services in high- risk zones	Annual EPI planning and review process Microplanning	Coordination of communication in high- risk zones			Job aids on messages for immunization (speaking book)	Integrated disease and response surveillance (polio, measles, yellow fever, neonatal tetanus)	Capacity building in RED, MLM training and IIP training on use of continuous temperature monitoring devices	
Nigeria DTP3 2013: 46% DTP3 2015: 56%	Engage military escort for vaccination activities in security-deprived north- east	Annual EPI planning and review process Micro-planning Identification of high risk communities and quarterly outreach to them Inclusion of previously missed populations using geographic information system data	Polio accountability framework adapted to RI with dashboards for RI In the states with Memorandum of Understanding (MOU) for RI, regular review meeting with all stakeholders. RI services integrated with other interventions (nutrition, water sanitation hygiene [WASH], birth registration)	IPV introduction, in particular in high- risk and insecure areas Use of “hit and run” to deliver immunization in conflict-affected areas	Monitoring of process and outcome indicators: cold chain, stocks, vaccination sessions, supervision, improvement plans based on supervision	Evidence generation through high-quality KAPB (Harvard Polling) New-born tracking, defaulter tracing and mobilization for RI Birth registration Nutritional screening and referral WASH promotion Using the village community mobilizers use of new technology and innovations to enhance demand and improve coverage	Visiting the polio high- risk sites Recording children immunized by settlements—linking micro plans with coverage. RI performance report using the DVD-MT and District Health Information System 2nd generation platforms Integrated disease and response surveillance (polio, measles, yellow fever, neonatal tetanus) Lassa fever, cholera, Ebola surveillance and response	Training of health facility and local government area staff Integrated primary health care training for health workers Training of EPI managers on effective supportive supervision	Emergency operations center used for EPI and emergency response (eg, Ebola) Tripartite MOU between state with Bill & Melinda Gates Foundation/ Dangote Foundation, and USAID, enabled accelerated RI improvements, supported by WHO and UNICEF
South Sudan DTP3 2013: 45% DTP3 2015: 31%	RI re-established in conflict zones	Annual EPI planning and review process Microplanning		Support to vaccination services Use of Rapid response missions to deliver immunization in 3 conflict-affected states	Supervision			Capacity building in immunization and epidemiology	
Afghanistan DTP3 2013: 70% DTP3 2015: 78%	Pilot project in districts (some high risk for polio)	Microplanning			Monitoring of sessions			Capacity building in RI	
Pakistan DTP3 2013: 72% DTP3 2015: 72%	Pilot project in districts (medium risk for polio)	Microplanning	Provincial and district review meeting		Monitoring of sessions	Evidence on knowledge, attitude, practices among parents and caregivers around RI through focus group discussions Social mobilization toolkit for RI		Capacity building in RI and communication	
Somalia DTP3 2013: 42% DTP3 2015: 42%	RI established in conflict zones with direct support of Global Polio Eradication Initiative partners	Annual EPI planning and review process	District review meetings	Delivery of vaccination services	Monitoring of process and outcome indicators: cold chain, stocks	Evidence generation through high-quality KAPB (Harvard Polling) Promote immunization Mobilize unvaccinated children	Data collection and analysis	MLM training for Ministry of Health and implementing partners	
India DTP3 2013: 83% DTP3 2015: 87%	Mission Indradanush: planning for RI intensification in high- risk zones	Annual EPI planning and review process Microplanning, including high- risk settlements	Block, district, state review meetings Accountability framework		Monitoring of process and outcome indicators at health facility level: vaccine stocks, sessions, supervision, coordination meetings held, RI coverage, reasons of nonvaccination	Evidence generation through high-quality KAPB (Harvard Polling) Newborn tracking RI due lists and defaulter tracing Promotion of family practices	Integrated disease surveillance and response	Capacity building of district health officers and health facility staff	

Abbreviations: DS, district; DTP3, 3 doses of diphtheria tetanus pertussis vaccine; DVD-MT, district vaccine and devices-management tool; EPI, Expanded Program for Immunization; HF, health Facility; ICC, Inter-Agency Coordination Committee; IIP, immunization in practice; KAPB, knowledge, attitude, practice behavior; MLM, midlevel management; NID, national immunization day; PIRI, periodic intensification of routine immunization; RED, Reaching Every District; RI, routine immunization; STOP, stop transmission of polio; WASH, water sanitation and hygiene.

### Policy and Strategy Development

In the domain of policy and strategy development, the polio eradication program’s expertise was used to prioritize activities to raise coverage in high-risk districts and include hard-to-reach communities in planning processes. The polio program used the routine immunization tools and explicitly focused on the areas with low coverage and high numbers of unimmunized children. Angola, Chad, Democratic Republic of Congo, and Ethiopia used the RED approach in polio high-risk districts. Chad introduced special vaccination strategies for Lake Chad’s island and nomadic populations using periodic intensification of routine immunization activities, integrating immunization activities with other interventions such as veterinary vaccination programs. In Democratic Republic of Congo, the *stratégie fluviale* (river strategy) was adapted from polio to routine immunization. In Democratic Republic of Congo, Nigeria, and South Sudan, negotiations with armed forces for access were organized for routine immunization, following the example of polio campaigns. In South Sudan, rapid response missions organized and coordinated by humanitarian organizations were used to deliver vaccines integrated with other interventions in 3 conflict-affected states. India developed a specific strategy to rapidly raise routine immunization coverage in high-risk zones using polio assets and lessons from successfully stopping poliovirus transmission (Mission Indradanush) that included intensification of routine immunization sessions targeting hard-to-reach populations, mobilization of eligible children and women for vaccination, and monitoring [14]. In Afghanistan and Pakistan, the strategies to use polio assets to strengthen routine immunization were piloted in a limited number of districts without major polio activities.

### Planning

The development of an annual national immunization coverage improvement plan encompassing broader immunization goals was 1 of the 3 output indicators for objective 2 of the PEESP [7]. This output was monitored by the Routine Immunization subgroup to the Immunization Management Group using 5 criteria: (1) annual EPI plans contain SMART (specific, measurable, achievable, relevant, and time-bound) objectives; (2) RED or Reaching Every Community activities are described in the plan, particularly focusing on high-risk districts; (3) the role and contribution of polio-funded staff and assets is clearly articulated; (4) the plan includes full costs of all components; and (5) there is evidence of approval by the Interagency Coordinating Committee or equivalent body. Although all 10 countries had an annual EPI plan, these differed considerably in their comprehensiveness, detail, feasibility, accuracy of costs, and political backing. In addition, the contribution of GPEI-funded personnel and assets in routine immunization strengthening was often vague and nonspecific. The Routine Immunization subgroup of the Immunization Management Group supported countries to develop their annual plans in 2014 and 2015. In 2014, 7 of the 10 countries’ plans met at least 3 of the 5 criteria. In 2015, it was 9 of the 10 country plans that did so, and in 2016, 6 of the 10 country plans met at least 3 criteria (see Table 2). Pakistan and Afghanistan’s annual planning was most challenging due to persistent wild polio virus circulation and parallel planning of polio eradication activities alongside broader immunization goals.

**Table 2.** Annual Expanded Program on Immunization Plans Encompassing Broader Immunization Goals for the 10 Focus Countries, 2014–2016

	Annual EPI plan 2014	Annual EPI plan 2015	Annual EPI plan 2016
Angola	5/5	5/5	3.5/5
Chad	5/5	5/5	5/5
DRC	4/5	3/5	4/5
Ethiopia	4/5	3/5	2/5
Nigeria	5/5	4/5	2/5
South Sudan	0/5	3/5	4/5
Afghanistan	3/5	4/5	2/5
Pakistan	0/5	0/5	2/5
Somalia	2/5	5/5	3/5
India	5/5	4/5	4/5

The five criteria on which they were evaluated were the following: (1) contains SMART (specific, measurable, achievable, relevant, and time-bound) objectives; (2) Reaching Every District or Reaching Every Community activities, with particularly focus on high-risk districts; (3) clearly articulates the role and contribution of polio-funded staff and assets; (4) accounts for the full costs of all components in the plan; and (5) provides evidence of approval by the Interagency Coordinating Committee (ICC) or equivalent body.

Microplanning for immunization at the operational level was supported in all focus countries by polio assets. In Nigeria, the polio program conducted an extensive geographic information system mapping exercise that revealed previously missed populations. Revised microplans for campaigns and routine immunization included these populations, and specific activities were planned for hard-to-reach populations. In addition, polio resources provide support to integrated routine immunization and maternal, new-born and child health (MNCH) services in 4000 hard-to-reach and underserved communities in 10 states of northern Nigeria [15]. In Democratic Republic of Congo, the polio program developed the *approche village* (village approach), which ensures that every village is included in the microplans, that was later applied to the RED approach in routine immunization.

### Management and Oversight

The management and oversight of the polio program has relied heavily on developing actionable metrics, collecting quality data, and analyzing information for action and decisions. This approach was applied to the routine immunization programs in Angola, Chad, Democratic Republic of Congo, Ethiopia, Nigeria, Pakistan, Somalia, and India. In Nigeria, the accountability framework that helped to strengthen polio program performance was adapted for routine immunization with dashboards on key process indicators, such as stock availability and vaccination sessions. With a focus on high-risk local government areas, the national and state emergency operations centers monitored the implementation of work plans and routine immunization sessions by health facility on a weekly basis and reviewed records on mobilization of children, defaulter tracing, and supportive supervision. In Somalia, regular district review meetings were instituted to enhance routine immunization implementation and raise coverage in high-risk areas in the Banadir region of Somalia’s South Central Zone and the Marrodjex and Sool regions of Somaliland.

### Implementation and Service Delivery

In most countries, implementation and service delivery for routine immunization was undertaken by regular government staff. However, in conflict areas in South Sudan and Somalia, polio-funded personnel and GPEI contractors were directly involved in routine immunization service delivery. In Nigeria, the accelerated introduction of IPV to rapidly increase population immunity in insecure areas and active transmission zones (Borno, Yobe, selected local government areas in Kano) was undertaken by polio assets, and identified key lessons were applied in the subsequent country-wide introduction of IPV into routine immunization [15].

### Monitoring

In the polio program, real-time monitoring mechanisms, such as independent monitoring and lot quality assessments, monitor process and outcome indicators before, during, and directly after campaigns and are used to adjust service delivery strategies to reach the very hard to reach. Routine immunization programs used this experience and introduced monitoring tools on processes and outcomes in Chad, Democratic Republic of Congo, Nigeria, Afghanistan, Pakistan, Somalia, and India that provided data on indicators to help raise coverage. In Chad, health facility–level RED indicators included availability of supplies (vaccines, devices, reporting tools), data quality, cold chain, capacity of health workers, vaccination sessions, children mobilized, and children immunized. These data were collected during health facility supervision visits and analyzed during review meetings. In Nigeria, real-time tracking of routine immunization supervision has started with polio resources. In this system, the supervisor sends key findings of his supervision visit, such as percentage of sessions conducted as compared with planned, availability of vaccine reporting tools at vaccination site, and vaccination status in sample communities by short message service (SMS) system to a phone number at the national level where data are analyzed and then sent back to the state. These data are discussed at the state level, and corrective measures are proposed. Throughout Somalia, polio-funded personnel are closely involved in all aspects of the routine immunization system, supporting the collection, transmission, and analysis of data, vaccine management, and monthly program reviews.

### Communication and Community Engagement

The communication and community engagement expertise area of the polio program was extended and adapted to routine immunization. The GPEI-supported Harvard Opinion Research Polling provides robust quantitative data on social issues such as polio eradication, and the approach was expanded to routine immunization in Democratic Republic of Congo, Nigeria, Pakistan, Somalia, and India [16–18]. Polio social mobilization networks were used for routine immunization defaulter tracing, newborn tracking, nutrition screening and referral, and promotion of family planning in Angola, Chad, Democratic Republic of Congo, Ethiopia, Nigeria, Pakistan, Somalia, and India. GPEI formalized and incentivized these efforts in some countries (Nigeria, Pakistan, and India). In other countries, GPEI improved capacity and coordination of communication/community engagement efforts (Chad, Ethiopia, and Somalia).

In India, the Social Mobilization Network (SMNet) engaged >8000 frontline social mobilizers, mostly female, in the states of Uttar Pradesh, Bihar, and West Bengal to advocate for vaccination in their own communities, which were underserved, marginalized, and at risk for polio outbreaks and other communicable diseases. The SMNet applied a range of community engagement approaches involving individuals, communities, religious leaders, and policy makers, creating champions of change in the most disadvantaged communities and reaching 2.7 million families each month in the 3 states. The approaches were based on evidence and addressed the beliefs and behavior for health at the individual level and determinants for health at the community level, focusing on perceptions and priorities. Although focusing initially on generating demand for polio vaccination, later the approaches promoted full infant immunization and other health and sanitation interventions related to maternal and children’s health, resulting in an increase in DTP3 coverage from 36% in 2009 to 78% in 2016 in Uttar Pradesh and from 54% to 89% in Bihar (WHO/UNICEF India, unpublished data). Elsewhere in this supplement, details are reported on the India experience [19]. Learning from the example of India, Nigeria’s polio community infrastructure has built demand for routine immunization through 11000 community mobilizers, mostly female, operating in high-risk areas or in hard-to-reach settlements and engaging with local religious and community leaders. Female mobilizers conduct regular household visits to educate families on key family practices, including immunization, hygiene promotion, and birth registration; conduct dialogues with communities; and track newborns and pregnancies with appropriate referral. This resulted in referral of >322000 newborns to routine immunization services in 2014; notification of communicable diseases, including acute flaccid paralysis (AFP), measles, and cholera; and referral of >32000 malnourished children to appropriate care. These efforts have helped to establish trust in the health system (Nigeria Ministry of Health, unpublished data).

In Ethiopia, polio-funded communication materials in book form with recorded messages (speaking books) are used by health promotors to deliver integrated immunization messages. In Chad, polio-funded communication personnel oriented community mobilizers on active house-to house search of defaulters from routine immunization and subsequent registration of all children aged <2 years in the community to mobilize those who have never been vaccinated, with monthly monitoring meetings to review performance, identify challenges, and agree on corrective activities for the next month. This has contributed to an approximate 2-fold increase in the number of children vaccinated with DTP3 (Ministry of Health, Chad, unpublished data).

In support of routine immunization, Pakistan’s polio program initiated a series of focus group discussions to gauge existing knowledge, attitudes, and practices of parents and caregivers toward routine immunization. In particular, the main barriers preventing parents and caregivers from bringing children to health facilities for routine immunization were explored. The findings of the focus group study enabled further fine-tuning of communication strategies and communication tools and pretesting of communication materials before mass production and roll out. This resulted in a well-developed, community-derived social mobilization kit for routine immunization.

### Surveillance Systems and Data Analysis

The improvement of surveillance systems and data analysis has been spearheaded by the polio program and is critical to improve health systems. In Angola, Chad, Democratic Republic of Congo, Ethiopia, Nigeria, and India, AFP surveillance was expanded into the Integrated Disease Surveillance and Response (IDSR) system, which includes measles, rubella, yellow fever, and neonatal tetanus [20, 21]. In Chad, Democratic Republic of Congo, and Nigeria, weekly visits to high-risk areas for AFP surveillance were extended to include surveillance for other vaccine-preventable diseases. In Democratic Republic of Congo and Nigeria, the polio surveillance systems also supported the cholera, Ebola, and Lassa fever outbreaks and responses. In Angola, polio-funded staff were instrumental to the investigation and response to the recent yellow fever outbreak. Polio personnel are further engaged in facilitating the e-warning system for natural disasters. Logistical support is provided for shipment of samples for testing for diseases such as measles, Lassa fever, and cholera. Capacity building for AFP surveillance incorporates IDSR modules at all levels.

In addition to surveillance, immunization performance data systems have been supported by the polio-funded infrastructure. In India, the national polio surveillance project strengthened routine systems through holding regular task force meetings at all levels, reviewing data and using data to improve performance, on vaccine stocks, sessions, and card checks of all antigens in infant immunization in high-risk communities. This approach has been replicated in Nigeria. In India and Nigeria, high-risk communities identified through the polio program were recorded by settlement, and this information was integrated into RI microplans. In Nigeria, the use of data systems such as District Vaccine & Devices-Management Tool (DVD-MT) and District Health Immunization Systems has been supported by polio-funded personnel. In Angola, immunization system data quality assessments have been supported by polio-funded personnel.

### Capacity Building

Polio-funded staff in all priority countries have supported the capacity building of national counterparts on various topics and in various fora (eg, midlevel management training of district managers and RED training across routine immunization programs). In Democratic Republic of Congo, stop transmission of polio (STOP) consultants have provided on-the-job training for district management teams in polio high-risks areas on the RED approach. In Pakistan, a series of trainings sessions was organized on social mobilization, including practical applications for frontline workers at the level of union councils. In Angola, polio-funded staff played a critical role in facilitating district trainings on RED strategy, local microplanning, data quality self-assessments, and supportive supervision of routine immunization at health facilities and outreach sites. In Ethiopia, polio-funded personnel supported RED training, capacity building using midlevel management training and immunization-in-practice modules, as well as training on the use of continuous temperature monitoring devices. In Somalia, training of communication networks included routine immunization promotion and referral of partially or unvaccinated children for all antigens. These routine immunization messages were also used during polio campaigns.

### Coordination and Partnerships

In the domain of coordination and partnerships, the GPEI-initiated emergency operations center in Nigeria was successfully used for the Ebola outbreak. In Democratic Republic of Congo, trans-border coordination of activities and mobilization of parliamentarians was extended to broader immunization goals. In addition, the interagency coordination committee (ICC) coordinated partners’ support for immunization and wider health. The GPEI leadership initiated a dialogue with Chad’s president and Nigeria’s state governors to enhance political leadership in immunization that has been expanded to immunization and wider health.

## CHANGES IN VACCINATION COVERAGE

Achieving improvements in immunization systems depends on multiple factors. Polio functional expertise in different areas can help strengthen certain components of immunization systems and outcomes (ie, increase vaccination coverage and reduce inequities in immunization), but other factors such as security, availability of vaccines and supplies, and quantity and quality of health work staff may impact the quality of immunization systems and the extent to which coverage and equity targets are achieved. As presented in Table 1, DTP3 coverage in 6 of the 10 focus countries improved between 2013 and 2015 (Chad, Democratic Republic of Congo, Ethiopia, Nigeria, Afghanistan, and India), coverage stayed the same in 2 countries (Somalia and Pakistan), and coverage dropped in 2 countries (South Sudan and Angola) [1]. In the 6 countries were coverage improved, reductions in the number of children who did not receive DTP3 between 2013 and 2015 were substantial: 1 million reduction in India, 0.6 million reduction in Nigeria, 0.4 million reduction in Ethiopia, 0.1 million reduction in Democratic Republic of Congo, and <100000 reduction in both Afghanistan and Chad [1]. In India, polio assets have helped increase coverage in the most challenging settings, vaccinating an additional 7.6 million children during the first phase of Mission Indradhanush [14] and contributing to the reduction of underimmunized children among the most disadvantaged children. In Angola, the applied polio expertise contributed to an approximate 10% increase in DTP3 coverage in 34 of the 35 focus districts between 2014 and 2015 (Angola administrative coverage reports, unpublished data). In Ethiopia, the combination of polio assets and other resources resulted in an increase from 31% to 48% in the proportion of high-risk zones having at least 80% DTP3 coverage. In Democratic Republic of Congo, systematic support, including polio assets for the RED approach, led to coverage improvements in target geographies (Democratic Republic of Congo administrative coverage reports, unpublished data). Although these reports are encouraging, validation of administrative coverage remains challenging, and coverage surveys have not been conducted to validate these changes.

## LESSONS FROM POLIO ERADICATION AND ROUTINE IMMUNIZATION

Although some studies suggest that polio eradication has a negative effect on immunization systems, particularly in countries with very weak health systems and with frequent immunization campaigns [8, 9], others conclude that polio eradication can have positive effects on routine immunization, particularly when system strengthening is taken into consideration during planning for polio activities [10, 11, 22–25].

The polio program has been globally driven, and country ownership has been challenging in some countries, leading to costly intensive support. Accountability frameworks (eg, in Nigeria) have been developed mainly by partners and mostly applied to partner agency personnel (Nigeria Ministry of Health, unpublished data). Hence lessons for countries remain to be learned. Plans have been fragmented and financial support earmarked for specific activities, putting country priorities in a backseat.

As a result of the global drive and, in the last few years, the global emergency to end polio, incentives have been provided to ensure the achievement of rapid results in polio eradication, potentially at the cost of sustainability and community ownership. This may have long-lasting negative effects because community volunteers now expect financial compensations for their activities in the communities. Performance of AFP surveillance has been boosted by compensation for the transport of stool samples, and other programs have taken advantage of available polio funding; in the future, surveillance of other diseases maybe challenged by the expectation of compensation.

Although India’s Mission Indradhanush has been lauded for its immediate results and for increasing coverage in vulnerable communities, the campaign mode has been questioned for its lack of sustainability. The government of India is looking to expand the approach to enhance sustainability—for example, by including stronger capacity building and including previously unreached communities in systematic microplans [26–28].

To counterbalance possible negative effects of vertical disease control programs, several studies conclude that eradication initiatives should define targets that include system strengthening [9, 11]. The PEESP intends to address some of these issues and defined 3 targets regarding system strengthening (comprehensive plans, time spent by polio-funded personnel on routine immunization strengthening, and increased DTP3 coverage in high-risk districts) and is attempting to create synergies between polio and routine immunization [2, 13].

Comprehensive country-driven and -owned operational annual EPI planning processes lead to improved coordination between partners and has a potential to better focus support to national priorities [29]. This approach has recently received global attention as a tool to harmonize partner support under the leadership of Ministry of Health national immunization programs. Moreover, Gavi, the Vaccine Alliance, has prioritized during its current strategic period (2016–2020) supporting the quality of annual EPI plans as the platform to consolidate all program assistance requirements and accelerate coverage improvements and reduction of the equity gap [30]. Importantly, the experiences of setting criteria of the annual EPI plans described in this article provide lessons for national immunization programs beyond the 10 polio focus countries, which can be leveraged by Gavi Alliance partners. The polio infrastructure has supported the implementation of new vaccine introduction and health systems’ strengthening efforts using Gavi alliance resources. The Gavi alliance has financed health systems’ strengthening in all 10 focus countries, supporting capacity building, infrastructure improvements (including immunization supply chain), and increasingly the implementation of the RED approach, in addition to new vaccine introduction, including hepatitis B vaccine, haemophilus influenza tybe B vaccine, pneumococcal conjugate vaccine, rotavirus vaccine, measles-rubella vaccine, and IPV [31].

Lessons learned in advancing global health security are illustrated through the Nigeria example, where the country’s national surveillance system and disease response successfully stopped a potential Ebola outbreak thanks to a well-functioning Emergency Operation Center supported by the polio infrastructure [32, 33].

In efforts to reduce equity gaps, the program introduced special strategies to reach underserved populations with OPV and other health services, including routine immunization, and special approaches for security-compromised areas, raising coverage and potentially reducing inequities in immunization. The program benefitted from polio expertise that strengthened community engagement, religious leaders’ and influencers’ engagement, surveillance, cold chain, and logistics. The accountability framework introduced by the polio program has been adopted by the broader immunization system and has shown positive signs of contributing to a reduction in the number of underimmunized children in Nigeria, with increase in national coverage from 46% to 56% from 2013 to 2015 [1].

Routine immunization systems have used the result-based approach from the polio program to focus on targeting high-risk areas and critical barriers. In addition, the real-time monitoring elements of the polio program have the potential to build accountable and strong immunization and health systems.

The evidence-driven vaccination strategies of the polio eradication program showcase the program’s expertise in reaching the most vulnerable and difficult-to-reach populations, bringing equity to the forefront. The “sanctuaries” where poliovirus continues to circulate or areas that are at risk for polio outbreaks are often also deprived of most primary health care. The increasing focus on people and communities, building trust between the caregivers and the program, and the realization that the interaction between the vaccinator and caregiver is a critical moment to enhance immunization demand helped contribute to progress on polio eradication, and this focus is now being applied to routine immunization in priority countries. The systematic evidence that the polio program has gathered on reasons for missed vaccination, social issues and trust, and in-process monitoring can be applied to routine immunization and wider MNCH programming to increase coverage [16]. The engagement of communities—in particular female community members—and traditional and religious influencers in the program has built trust in the system and created demand for immunization and other services. Female community members also promote healthy behaviors and birth registration. In a sense, the vertical polio program has made the full circle back to a more comprehensive primary healthcare approach, where communities are at the center [16–18].

Although polio eradication is a global public good and global steering is justified, successful interruption of polio virus circulation hinged on a coherent program under strong government leadership, as shown in India [26]. Routine immunization is foremost a government-driven program, and protecting children and women from all vaccine-preventable diseases is a human right and a government responsibility. Building routine systems led by governments may be slow, but ultimately will be sustainable, as local solutions will guide the way to better outcomes. Community partnership and strong accountability systems are critical for all programs, and polio eradication has shown a pathway for equitable immunization programs to achieve universal coverage.

## Notes

***Disclaimer.*** The findings, interpretations and conclusions expressed in this paper are those of the authors and do not necessarily reflect the policies or views of the organizations with whom the authors are affiliated.

***Supplement sponsorship.*** This work is part of a supplement coordinated by the Task Force for Global Health with funding provided by The Bill and Melinda Gates Foundation and the Centers for Disease Control and Prevention.

***Potential conflicts of interest.*** All authors: No reported conflicts. All authors have submitted the ICMJE Form for Disclosure of Potential Conflicts of Interest. Conflicts that the editors consider relevant to the content of the manuscript have been disclosed.
